# Systemic hematologic, metabolic, and inflammatory dysregulation in liver cirrhosis and hepatocellular carcinoma: diagnostic utility of novel indices in an Egyptian cohort

**DOI:** 10.1186/s12876-026-04752-2

**Published:** 2026-04-13

**Authors:** Dalia E. Sherief, Hadeer Hamdy Shehata, Nahla Nosair, Amira A. A. Othman, Mohamed W. Saleh, Emad Sadaka, Rasha Elgamal

**Affiliations:** 1 Faculty of Medicine, Clinical Pathology, Kafr Elsheikh University, Kafr Elsheikh, 33511 Egypt; 2 Department of Internal Medicine, Suez University, Suez, 43511 Egypt; 3 Department of Clinical Oncology and Nuclear Medicine, Faculty of Medicine, Kafr Elsheikh University, Kafr Elsheikh, 33511 Egypt; 4 Faculty of Medicine, Clinical Pathology, Rasha Elgamal, Suez University, Suez, Suez, 43511 Egypt; 5 Department of Internal Medicine, Faculty of Medicine, Suez University, Suez, Egypt

**Keywords:** Hepatocellular Carcinoma, Liver Cirrhosis, Systemic Inflammation Response Index, GGT-to-Lymphocyte Ratio, Liver Function Tests, Metabolic Dysregulation, Inflammatory Biomarkers, Egypt

## Abstract

**Background:**

The transition from chronic liver disease to cirrhosis and ultimately hepatocellular carcinoma (HCC) is accompanied by progressive systemic alterations involving hematologic, metabolic, and inflammatory pathways. Understanding these interconnected changes may refine disease characterization and support early diagnostic or prognostic stratification, especially in high-burden regions such as Egypt. This study aimed to delineate the comprehensive clinical, hematologic, metabolic, and inflammatory profiles distinguishing Egyptian patients with HCC and liver cirrhosis from healthy controls, and to evaluate the diagnostic and discriminative utility of novel systemic inflammatory indices.

**Methods:**

In this comparative cross-sectional study, 211 participants were enrolled: 70 with HCC, 71 with HCV-related cirrhosis, and 70 healthy controls. Clinical, biochemical, and hematologic parameters were measured, including liver and renal function, lipid profile, and alpha-fetoprotein (AFP). Composite inflammatory indices were calculated: Systemic Inflammation Response Index (SIRI = neutrophils × monocytes/lymphocytes) and GGT-to-Lymphocyte Ratio (GLR = GGT / lymphocytes). Group comparisons were performed using non-parametric tests. Correlations between indices and clinical or tumor features were analyzed by Spearman’s coefficient. Diagnostic performance was assessed using ROC curve analysis and multivariable logistic regression adjusted for age and sex.

**Results:**

Both patient groups demonstrated marked systemic dysregulation relative to controls. Hemoglobin, platelet count, albumin, and HDL-C were significantly reduced, whereas bilirubin, transaminases, ALP, INR, AFP, and insulin were progressively elevated (all *P* < 0.001). Gamma-glutamyl transferase (GGT) was significantly elevated in both patient groups compared to controls, with the highest levels observed in the HCC group. The inflammatory indices SIRI and GLR were significantly higher in cirrhosis and HCC (median SIRI: 1.25 vs. 1.2 vs. 0.6; *P* < 0.001). GLR, in particular, demonstrated strong discriminatory performance for distinguishing HCC from cirrhosis (AUC = 0.881; 95% CI 0.842–0.921; *P* < 0.001), achieving significantly higher diagnostic accuracy than SIRI (AUC = 0.742). Subgroup analysis confirmed its superior diagnostic accuracy, especially in patients with advanced liver dysfunction (Child-Pugh B/C; AUC = 0.92). Furthermore, a high GLR (> 22.5) was an independent predictor of reduced overall survival in the HCC cohort (adjusted HR = 2.95; *P* = 0.004). In multivariable models, elevated GLR and low albumin independently predicted HCC presence (adjusted ORs 2.31 and 0.54, respectively; *P* < 0.01).

**Conclusion:**

Distinct and progressively severe hematologic, metabolic, and inflammatory disturbances characterize the continuum from cirrhosis to HCC. The Gamma-Glutamyl Transferase-to-Lymphocyte Ratio (GLR) showed a strong association with the presence of HCC and demonstrated strong discriminatory performance (AUC = 0.881) for its differentiation from cirrhosis, suggesting it is a promising candidate worthy of further validation in surveillance programs for high-risk Egyptian patients.

## Introduction

Hepatocellular carcinoma (HCC) is a leading cause of cancer-related mortality worldwide, particularly in regions with a high burden of chronic liver disease [1]. Globally, more than 80% of HCC cases develop in the setting of hepatic cirrhosis, a final common pathway of persistent liver injury, inflammation, and fibrosis [2]. Egypt has long experienced one of the world’s highest prevalences of hepatitis C virus (HCV) infection, estimated at 6–7% of the adult population, which has led to a correspondingly high incidence of cirrhosis and HCC despite national antiviral treatment campaigns [3, 4]. Understanding the systemic alterations that accompany the transition from cirrhosis to malignancy in this high-risk population remains a major clinical and public health priority.

The pathogenesis of HCC is not confined to hepatocytes alone but reflects a multisystemic metabolic–inflammatory dysregulation. Chronic hepatic inflammation triggers cytokine cascades, oxidative stress, and immune cell activation, which extend beyond the liver and reshape systemic homeostasis [5]. Clinically, this manifests as derangements in hematologic parameters, coagulation balance, and lipid metabolism, accompanied by subclinical insulin resistance and metabolic remodeling [6, 7]. These changes are not only consequences of hepatic dysfunction but may also contribute to disease progression, promoting a pro-oncogenic microenvironment and impaired immune surveillance [8].

Routine biochemical and hematologic indices have long been used to evaluate liver disease severity through tools such as the Child–Pugh and MELD scores. However, these traditional parameters capture only limited aspects of the broader inflammatory–metabolic crosstalk that characterizes hepatocarcinogenesis [9]. Recent evidence has highlighted the value of composite inflammatory indices, derived from routine blood counts, as surrogate markers of the systemic inflammatory state in cancer [10]. The Systemic Inflammation Response Index (SIRI), calculated from neutrophil, monocyte, and lymphocyte counts, integrates both innate and adaptive immune responses and has shown prognostic relevance in diverse malignancies, including HCC [11, 12]. SIRI was selected for this head-to-head comparison because it incorporates monocytic activity in addition to the neutrophil-lymphocyte balance captured by simpler indices (e.g., NLR), potentially offering a more comprehensive reflection of systemic inflammation. Similarly, the Gamma-Glutamyl Transferase-to-Lymphocyte Ratio (GLR) combines a sensitive hepatic enzyme linked to oxidative stress with an immune parameter, providing an accessible reflection of the inflammatory–metabolic axis [13, 14]. We hypothesized that GLR, by integrating a direct marker of hepatic stress (GGT), would provide superior discrimination in the context of liver disease progression compared to SIRI.

While several studies have explored individual inflammatory markers or biochemical abnormalities in HCC, few have comprehensively compared the full spectrum of hematologic, metabolic, and inflammatory alterations across the continuum from health to cirrhosis to cancer within a single, well-characterized Egyptian cohort. Most prior Egyptian studies have focused on either tumor biomarkers such as AFP [15] or molecular markers such as circulating microRNAs [16], leaving a gap in understanding the systemic physiological context in which these molecular events occur.

Therefore, this study aimed to perform an integrative, systemic analysis of the progressive clinical, biochemical, and inflammatory alterations across healthy, cirrhotic, and HCC subjects in an Egyptian population. Moving beyond the reporting of isolated parameter changes, we focused on novel composite indices. We specifically hypothesized that the Gamma-Glutamyl Transferase-to-Lymphocyte Ratio (GLR), as an index integrating a direct marker of hepatic stress (GGT) with an immune parameter (lymphocyte count), would provide superior discrimination between cirrhosis and HCC compared to indices derived solely from blood cell counts, such as the Systemic Inflammation Response Index (SIRI). This hypothesis tests the premise that a biomarker reflecting the hepatic-immune axis is more relevant to liver disease progression than generic systemic inflammation. By elucidating these systemic patterns, this study seeks to contribute a broader pathophysiologic framework for liver disease progression and to identify accessible, low-cost indicators that could complement established biomarkers in clinical practice for high-risk populations.

## Subjects and methods

### Study population and design

This prospective, observational case–control study was conducted at the Clinical Pathology and Internal Medicine Departments of Kafr Elsheikh University Hospitals, in collaboration with the Internal Medicine Department of Suez University Hospital, Egypt. The study period extended from June 2022 to March 2024. The research was designed to characterize systemic biochemical and inflammatory alterations across the disease spectrum from health to cirrhosis to hepatocellular carcinoma (HCC) within a pathophysiologically homogeneous cohort. To clearly attribute biomarker performance to disease stage rather than to confounding by divergent etiologic mechanisms (e.g., HBV, MASLD, alcohol), enrollment was restricted to patients with HCV-related disease. This design provides an integrative clinical–inflammatory profile of liver disease progression along a single, well-defined pathogenic pathway in an Egyptian population.

A total of 211 participants were enrolled and stratified into three groups representing progressive stages of liver disease: 70 patients with newly diagnosed, treatment-naïve HCC, 71 patients with HCV-related liver cirrhosis without radiologic or serologic evidence of malignancy, and 70 healthy controls with no history or biochemical evidence of hepatic or systemic disease. Healthy controls were selected using frequency matching to the overall case groups (HCC and cirrhosis) in a 1:1:1 ratio based on sex and age (within ± 5 years). This matching was performed prospectively during recruitment to ensure baseline comparability. Controls were recruited from the local community via health awareness campaigns and had no clinical or biochemical evidence of liver disease.

The diagnosis of HCC was confirmed by triphasic computed tomography (CT) or histopathological examination according to the American Association for the Study of Liver Diseases (AASLD) guidelines [17]. The diagnosis of cirrhosis was established by compatible clinical features, ultrasonographic findings, and biochemical abnormalities.

Sample size was determined a priori using G*Power version 3.1. Based on preliminary data and prior Egyptian studies evaluating inflammatory indices in HCC [18], we anticipated a moderate effect size for differences in GLR across groups. A standardized effect size (Cohen’s f) of 0.35 was therefore assumed. Using a one-way ANOVA framework (three groups: controls, cirrhosis, HCC), with a two-sided α of 0.05 and 80% power (1 − β), the required sample size was 60 participants per group. To compensate for potential exclusions or incomplete data, we increased the target sample size by 10–15%, resulting in a final enrollment of 70 participants per group (total *N* = 210). Our final enrollment of 211 participants met this target. This a priori calculation ensured the study was adequately powered for its primary diagnostic comparisons. The subsequent statistical robustness of our key results (e.g., high AUCs, significant multivariate models) confirms the analytical sufficiency of this sample size.

### Ethical considerations

This study received approval from the Medical Research Ethics Committee of Kafr Elsheikh University (Reference No. KFSIRB200-817). Written informed consent was obtained from all participants before enrolment. The study was conducted in accordance with the ethical principles of the Declaration of Helsinki (2013 revision) and complied with the ethical standards of the Egyptian Ministry of Health research regulations.

### Inclusion and exclusion criteria

Eligible participants were adults aged between 18 and 75 years. HCC cases included only those who were newly diagnosed and had not yet received surgical, locoregional, or systemic therapy for HCC. To avoid confounding from the rapid biochemical and immunological shifts associated with viral clearance, patients who had received direct-acting antiviral (DAA) therapy within the preceding 12 months were explicitly excluded. It is essential to note that this exclusion criterion resulted in our study population consisting entirely of DAA-naïve patients or those with a remote treatment history (more than 12 months). The effects of recent DAA therapy on the inflammatory, hematologic, and metabolic parameters under investigation were therefore not assessed in this study. Cirrhotic patients were required to have documented chronic HCV infection and imaging evidence of cirrhosis, while controls had to demonstrate normal laboratory and imaging findings.

Exclusion criteria encompassed any concurrent bacterial or viral infection, autoimmune liver disease, significant alcohol consumption (defined as > 30 g/day for men and > 20 g/day for women), or a clinical diagnosis of alcohol-related liver disease or non-HCV-related etiologies of cirrhosis to maintain etiologic homogeneity. Patients with previous or concurrent malignancies other than HCC, those with renal insufficiency (estimated glomerular filtration rate < 60 mL/min/1.73 m²), or known hematologic disorders were excluded to avoid confounding effects on inflammatory and hematologic indices. Participants using medications known to significantly influence liver enzymes, inflammatory markers, or lipid profiles, including but not limited to systemic corticosteroids, immunosuppressants, antiepileptic drugs (e.g., phenytoin, carbamazepine), statins, or direct-acting antiviral (DAA) therapy for HCV (within the preceding 12 months) were also excluded. This DAA-related exclusion criterion is particularly important to highlight, as DAA therapy induces rapid virologic clearance and significant immunological reconstitution that could independently alter lymphocyte counts, inflammatory indices, and liver function tests, thereby confounding the disease-specific signals we aimed to capture. We therefore emphasize that patients with recent DAA exposure were intentionally not included to preserve internal validity. Furthermore, patients with active smoking habits, a known diagnosis of diabetes mellitus, or metabolic syndrome were excluded to minimize confounding from these metabolic and inflammatory risk factors. This was critically important to avoid confounding effects on the primary inflammatory and hematologic indices under investigation, as chronic kidney disease is independently associated with lymphopenia, thrombocytopenia, and elevated GGT levels, which would directly alter the calculation and interpretation of both SIRI and GLR. Additional exclusions included recent major surgery, trauma, or blood transfusion within three months, as well as chronic corticosteroid or immunosuppressive drug use, all of which could significantly alter systemic inflammatory responses. These criteria were applied uniformly across all study groups to ensure internal validity and comparability of the results.

### Clinical and laboratory assessment

#### Clinical evaluation

All patients underwent comprehensive clinical evaluation, including history taking, physical examination, and documentation of ascites, jaundice, and hepatic encephalopathy. The severity of liver disease was graded using the Child–Pugh classification [19]. For HCC patients, tumor size, number, and vascular invasion were recorded from radiologic reports. For subsequent analyses, ‘High Tumor Burden’ was defined as the presence of multiple tumors, macrovascular invasion, or a maximum tumor diameter ≥ 5 cm.

#### Laboratory assessment

##### Blood sampling and processing

Venous blood samples (5 mL) were drawn from each participant following an overnight fast. Serum was separated by centrifugation at 2000 × g for 10 min and analyzed immediately or stored at − 80 °C until testing.

Hematologic parameters, including hemoglobin (Hb), total leukocyte count (TLC), and platelet count, were measured using an automated hematology analyzer (Sysmex XN-1000, Japan). Liver function tests, alanine aminotransferase (ALT), aspartate aminotransferase (AST), alkaline phosphatase (ALP), gamma-glutamyl transferase (GGT), total and direct bilirubin, and serum albumin, were performed using an automated chemistry analyzer (Cobas 6000, Roche Diagnostics, Germany).

Renal function (creatinine) and lipid profile (total cholesterol, triglycerides, and high-density lipoprotein cholesterol [HDL-C]) were measured using enzymatic colorimetric methods. Prothrombin time (PT) was converted to the international normalized ratio (INR). Serum alpha-fetoprotein (AFP) was quantified by chemiluminescent microparticle immunoassay on the Abbott Architect i1000SR system, and serum insulin was measured by electrochemiluminescence on a Roche Cobas e411 analyzer.

##### Inflammatory indices calculation

Systemic inflammatory indices were computed from routine laboratory results: (1) Systemic Inflammation Response Index (SIRI) = (absolute neutrophil count [×10⁹/L] × absolute monocyte count [×10⁹/L]) / absolute lymphocyte count [×10⁹/L] [11]. (2) Gamma-Glutamyl Transferase-to-Lymphocyte Ratio (GLR) = GGT [U/L] / absolute lymphocyte count [×10⁹/L] [13]. Lymphocyte counts were absolute values (not percentages) expressed in cells ×10⁹/L, ensuring dimensional consistency in the ratio. Both indices were evaluated as continuous variables and also categorized according to optimal ROC-derived cut-offs.

### Outcome measures

The primary outcome of the study was to compare systemic inflammatory indices, specifically the Systemic Inflammation Response Index (SIRI) and Gamma-Glutamyl Transferase-to-Lymphocyte Ratio (GLR), among the three study groups and to evaluate their diagnostic capacity in differentiating HCC from cirrhosis.

Secondary outcomes included the assessment of progressive alterations in hematologic, biochemical, and metabolic parameters across the disease continuum; exploration of correlations between inflammatory indices and conventional biochemical or clinical variables such as Child–Pugh class and alpha-fetoprotein (AFP); identification of independent predictors of HCC using multivariable logistic regression models adjusted for relevant covariates; and determination of optimal diagnostic thresholds for SIRI and GLR using receiver operating characteristic (ROC) curve analysis.

### Statistical analysis

Statistical analyses were performed using IBM SPSS Statistics version 25.0 (IBM Corp., Armonk, NY, USA) and MedCalc version 22.017 (MedCalc Software Ltd., Ostend, Belgium). Data normality was assessed by the Shapiro–Wilk test. Continuous variables were expressed as median (interquartile range [IQR]) and compared among groups using the Kruskal–Wallis test, followed by Dunn–Bonferroni post-hoc analysis for pairwise comparisons. Categorical data were summarized as frequencies and percentages and analyzed using the Chi-square or Fisher’s exact test, as appropriate. Correlations between inflammatory indices and clinical or biochemical parameters were examined using Spearman’s rank correlation coefficient (ρ).

The diagnostic performance of the inflammatory indices and relevant biochemical markers was assessed through receiver operating characteristic (ROC) curve analysis. Optimal thresholds were determined using the Youden index [20], and differences between correlated areas under the curve (AUCs) were evaluated using the DeLong method [21].

Candidate variables were first screened using univariate logistic regression, with a liberal significance threshold of *p* < 0.10 to avoid excluding potentially important predictors (Table 7). All variables significant at this level were considered for multivariable modeling. The final model was constructed following a pre-specified, parsimony-driven, and pathophysiology-guided strategy rather than automatic inclusion of all univariate predictors. This approach aimed to ensure model stability, avoid overfitting, and enhance clinical interpretability by selecting a minimal set of variables representing distinct, non-redundant biological domains relevant to HCC pathogenesis.

Variable selection was based on three principles: (1) representation of key pathophysiological domains (tumor burden, inflammation/immunity, hepatic function, metabolism); (2) avoidance of collinearity; and (3) preservation of an adequate events-per-variable (EPV) ratio. Consequently, markers of hepatocellular injury/cholestasis (AST, bilirubin) and the composite Child-Pugh score were excluded due to conceptual overlap and collinearity with core domains already represented (e.g., albumin for hepatic function, GLR for hepatic/immune stress). Including them would not provide unique independent information while compromising parsimony.

The final variables were therefore: GLR (inflammatory-immune-metabolic axis), AFP (tumor burden), albumin (hepatic synthetic function), and HDL-C (metabolic dysregulation). The inclusion of HDL-C was specifically motivated by its strong inverse correlation with systemic inflammation in our cohort (Table 6) and its established pathophysiological role. This selection resulted in a stable model with an EPV of 17.5. The final model’s calibration was assessed using the Hosmer–Lemeshow goodness-of-fit test, and its discriminative ability was quantified by the AUC. Internal validation was performed using bootstrap resampling with 500 iterations, a commonly used and statistically sufficient number for this purpose, to estimate optimism-corrected performance metrics and assess potential overfitting.

For survival analysis, follow-up time was calculated from the date of HCC diagnosis to the date of death or last clinical contact, whichever came first. Patients alive at the last follow-up were censored. Median follow-up time was estimated using the reverse Kaplan–Meier method. Overall survival was analyzed using Kaplan–Meier curves with the log-rank test. Cox proportional hazards models were used for multivariable adjustment, with results expressed as hazard ratios (HRs) and 95% confidence intervals (CIs).

Graphical representations, including boxplots, ROC curves, and correlation matrices, were generated using R software version 4.3.2 (R Foundation for Statistical Computing, Vienna, Austria) with the ggplot2 and pROC packages. A two-tailed P value < 0.05 was considered statistically significant.

## Results

### Demographic and clinical characteristics

A total of 211 participants were enrolled and stratified into three groups: hepatocellular carcinoma (HCC; *n* = 70), liver cirrhosis (*n* = 71), and healthy controls (*n* = 70). The study groups were well-matched at baseline, with no significant differences in age (*p* = 0.387) or sex distribution (*p* = 0.459) (Table 1).

**Table 1:** Baseline demographic characteristics of the studied groups

Characteristic	HCC (*n* = 70)	Liver Cirrhosis (*n* = 71)	Controls (*n* = 70)	*p*-value
Sex, *n* (%)				0.459
Female	24 (34.3)	28 (39.4)	31 (44.3)	
Male	46 (65.7)	43 (60.6)	39 (55.7)	
Age (years), Median (IQR)	64 (58–69)	62 (57–67)	63 (57–68)	0.387

Data are presented as *n* (%) or median (interquartile range). Comparisons among the three groups: sex distribution was compared using the Chi-square test; age was compared using the Kruskal–Wallis test. The groups were matched to ensure homogeneity in baseline demographics

Clinical assessment revealed a significantly different profile of liver disease severity between the two patient groups (Table 2). The cirrhotic group had a significantly higher proportion of Child-Pugh class C severity (28.2% vs. 14.3%, *p* = 0.045), driven primarily by worse coagulopathy (higher INR) and a higher prevalence of hepatic encephalopathy, as detailed in the component analysis in Table 2. These deficits in synthetic function and neurological status, which carry substantial weight in the Child-Pugh score, overcompensated for their less severe ascites, resulting in a higher overall classification of severity. This highlights that the diagnosis of HCC can occur in patients with moderately preserved synthetic function, underscoring the need for biomarkers independent of conventional severity scores.

**Table 2:** Clinical Characteristics and Child-Pugh Components of Cirrhosis and HCC Patients

Characteristic	HCC (*n* = 70)	Liver Cirrhosis (*n* = 71)	*p*-value
Ascites, *n* (%)			< 0.001
None	49 (70.0)	53 (74.6)	
Mild	12 (17.1)	16 (22.5)	
Severe	9 (12.9)	2 (2.8)	
Hepatic Encephalopathy, *n* (%)			0.018
None	65 (92.9)	57 (80.3)	
Grade 1–2	5 (7.1)	14 (19.7)	
INR, Median (IQR)	1.2 (0.3)	1.4 (0.5)	0.005
Albumin (g/dL), Median (IQR)	3.15 (0.6)	3.3 (0.6)	0.112
Total Bilirubin (mg/dL), Median (IQR)	2.7 (1.4)	1.9 (0.9)	< 0.001
Child–Pugh Class, *n* (%)			0.045*
A	47 (67.1)	39 (54.9)	
B	13 (18.6)	12 (16.9)	
C	10 (14.3)	20 (28.2)	

Data are presented as *n* (%) or median (interquartile range). *P*-values for categorical variables (Ascites, Encephalopathy, Child–Pugh Class) were derived from the Chi-square test. For continuous variables (INR, Albumin, Total Bilirubin), the Mann–Whitney U test was used for the direct two-group comparison between HCC and cirrhosis patients. The *p*-value for Child–Pugh Class (0.045*) reflects a post-hoc pairwise comparison (HCC vs. Cirrhosis) following a significant overall Chi-square test

### Hematologic, biochemical, and metabolic profiles

**Fig. 1:**
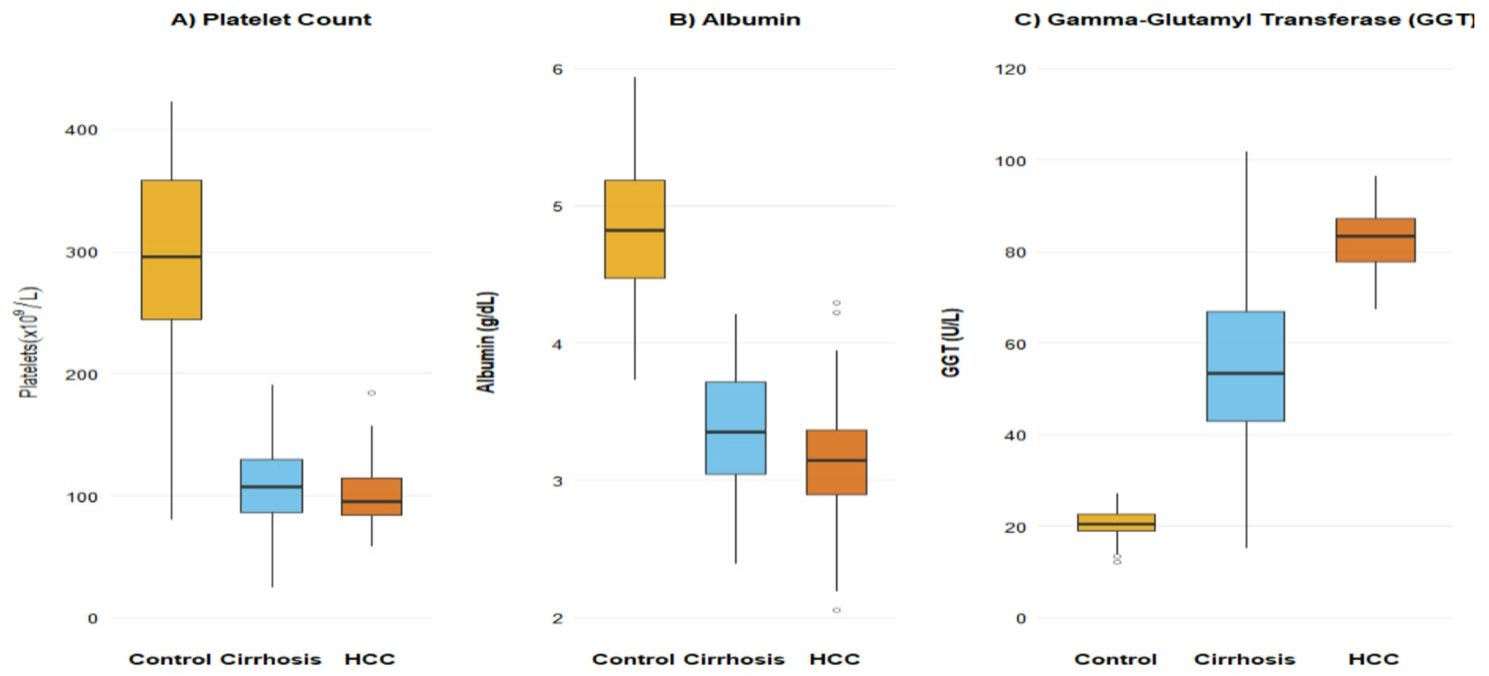
Progressive dysregulation of Hematologic, Metabolic, and Inflammatory Parameters. Boxplots illustrating the progressive alterations in key parameters across the disease spectrum (Control [n = 70], Cirrhosis [n = 71], HCC [n = 70]). **A** Platelet count (×10⁹/L) shows a marked decline in patient groups. **B** Albumin (g/dL) demonstrates a graded decrease from controls to cirrhosis to HCC. **C** Gamma-Glutamyl Transferase (GGT) shows elevated levels in both patient groups, with the highest median value in HCC (84 U/L), followed by cirrhosis (55 U/L). Boxes represent the interquartile range (IQR), the line inside the box is the median, and whiskers extend to 1.5×IQR

Systemic dysregulation was evident across all measured domains when comparing patient groups to controls (Table 3; Fig. 1). Hematologic analysis revealed expected features of advanced liver disease, including significantly lower hemoglobin levels and profound thrombocytopenia in both HCC and cirrhosis groups compared to controls (*p* < 0.001), with white blood cell counts being lowest in the HCC group (*p* = 0.005). Notably, absolute lymphocyte counts showed a significant stepwise decrease from controls to cirrhosis to HCC, with the lowest median value observed in the HCC group (1.2 × 10⁹/L; *p* < 0.001).

**Table 3:** Comprehensive hematologic, biochemical, and metabolic profiles of the study groups

Parameter	HCC (*n* = 70)	Cirrhosis (*n* = 71)	Control (*n* = 70)	*p*-value
Hemoglobin (g/dL)	10.2 (2.15)	9.8 (2.0)	12.7 (1.13)	< 0.001
WBC (×10⁹/L)	6.5 (4.1)	7.3 (4.4)	7.5 (2.7)	0.005
Lymphocytes (×10⁹/L)	1.2 (0.5)	1.5 (0.6)	2.1 (0.7)	< 0.001
Platelets (×10⁹/L)	100 (35)	110 (50)	313 (160)	< 0.001
ALT (U/L)	54.5 (34.3)	44 (11.5)	33 (4.3)	< 0.001
AST (U/L)	65 (19.8)	42 (10)	20 (3)	< 0.001
GGT (U/L)	84 (8)	55 (26)	20 (4)	< 0.001
ALP (U/L)	84 (13)	144 (36)	49.5 (12)	< 0.001
Total bilirubin (mg/dL)	2.7 (1.4)	1.9 (0.9)	0.5 (0.1)	< 0.001
Direct bilirubin (mg/dL)	1.8 (1.1)	1.0 (0.9)	0.2 (0.2)	< 0.001
Albumin (g/dL)	3.15 (0.6)	3.3 (0.6)	4.9 ^1^ (0.9)	< 0.001
INR	1.2 (0.3)	1.3 (0.5)	1.0 (0.03)	< 0.001
Total cholesterol (mg/dL)	130 (60)	125 (50)	156 (18)	< 0.001
Triglycerides (mg/dL)	165 (95)	120 (101)	101 (8)	< 0.001
HDL-C (mg/dL)	39 (10)	38 (9)	47 (4)	< 0.001
Creatinine (mg/dL)	1.2 (0.6)	1.02 (0.5)	0.8 (0.21)	< 0.001
AFP (ng/mL)	127 (126.3)	30 (37)	2.85 (1.3)	< 0.001
Serum insulin (µIU/mL)	19 (7)	21 (5)	7.4 (2.0)	< 0.001

Data are presented as median (interquartile range). *P-*values derived from the Kruskal–Wallis test for overall group comparisons. Pairwise post-hoc comparisons were performed using the Dunn–Bonferroni method, where the overall test was significant (*p* < 0.05)

*Abbreviations*: *WBC* white blood cell count, *ALT* alanine aminotransferase, *AST* aspartate aminotransferase, *GGT* gamma-glutamyl transferase, *ALP* alkaline phosphatase, *INR* international normalized ratio, *HDL-C* high-density lipoprotein cholesterol, *AFP* alpha-fetoprotein

^1^Albumin reference range for the laboratory: 3.5–5.2 g/dL

A pattern of progressive hepatic injury and dysfunction was observed. Liver enzymes (ALT, AST, ALP) and bilirubin showed a stepwise increase from controls to cirrhosis to HCC (all *p* < 0.001). Gamma-glutamyl transferase (GGT) was markedly elevated in both patient groups compared to controls, with the highest median value in the HCC group (84 U/L), followed by the cirrhosis group (55 U/L). Critically, as shown in Table 4, the Gamma-Glutamyl Transferase-to-Lymphocyte Ratio (GLR) was significantly higher in HCC than in cirrhosis. This demonstrates that the discriminatory power of GLR is derived from the integration of GGT with the marked and progressive lymphopenia observed in the HCC cohort, reflecting a combined hepatic and immune dysfunction signature. Synthetic liver function deteriorated accordingly, with serum albumin demonstrating a graded decline and INR showing a progressive increase (*p* < 0.001).

**Table 4:** Inflammatory indices among study groups

Index	HCC (*n* = 70)	Cirrhosis (*n* = 71)	Control (*n* = 70)	*p*-value
SIRI	1.25 (1.08)	1.20 (0.75)	0.60 (0.50)	< 0.001
GLR	30.0 (12.1)	15.0 (11.0)	4.8 (2.3)	< 0.001

Data are presented as median (interquartile range). *P*-values derived from the Kruskal–Wallis test for overall group comparisons. Post-hoc pairwise comparisons were performed using the Dunn–Bonferroni method. For SIRI, HCC vs. Cirrhosis was not significant (adjusted *p* = 0.42); all other pairwise comparisons were significant (adjusted *p* < 0.05). For GLR, all pairwise comparisons were significant (adjusted *p* < 0.05)

*Abbreviations*: *SIRI* Systemic Inflammation Response Index, *GLR* Gamma-Glutamyl Transferase-to-Lymphocyte Ratio

GLR was calculated as GGT/lymphocyte count for each participant before computing group medians; reported values therefore represent medians of the ratio distribution rather than ratios of group medians

Marked metabolic disturbances were also present. The lipid profile was increasingly atherogenic along the disease spectrum, with total cholesterol and HDL-C significantly depressed in patient groups, while triglycerides were highest in the HCC cohort (*p* < 0.001). Furthermore, serum insulin was markedly elevated in both patient groups, indicating significant insulin resistance. As anticipated, AFP levels were profoundly higher in HCC patients compared to both other groups (*p* < 0.001), underscoring the combined impact of hepatocellular injury and tumor burden.

### Inflammatory indices (SIRI and GLR) and inter-group comparisons

**Fig. 2:**
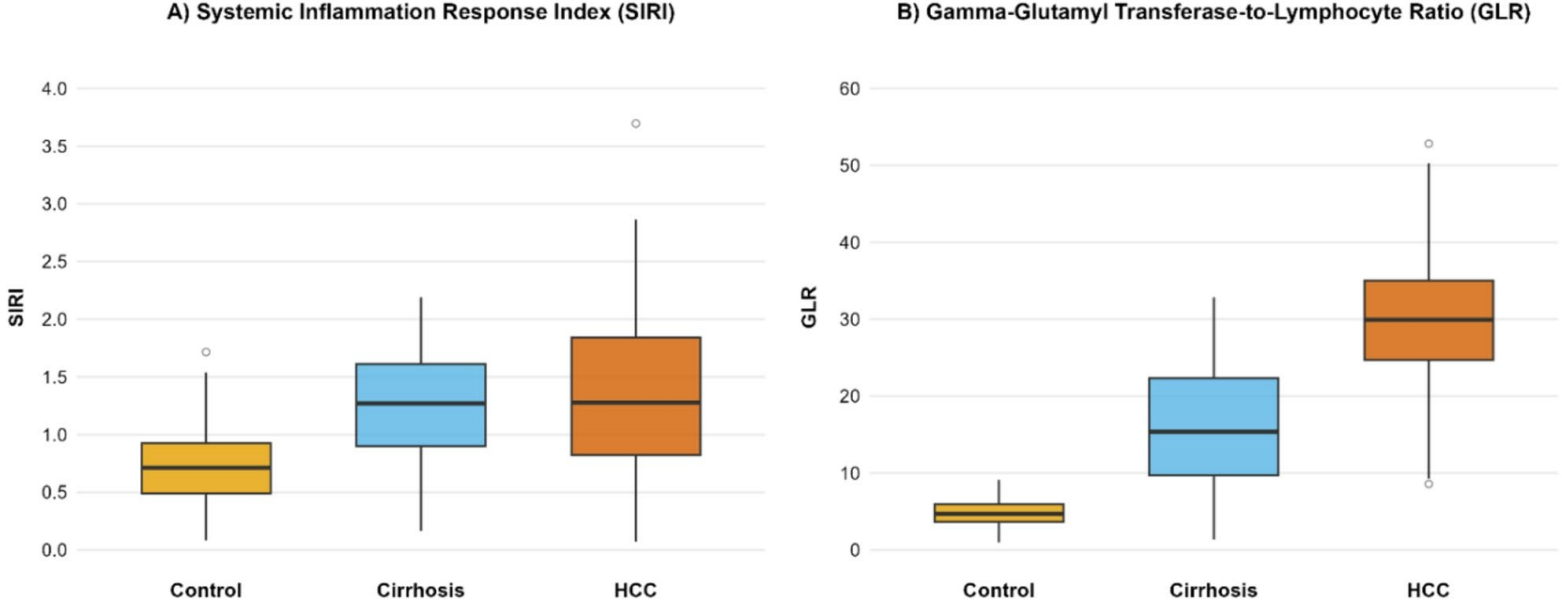
Comparative analysis of systemic inflammatory indices across the disease spectrum. Oxplots comparing the Systemic Inflammation Response Index (SIRI) and the Gamma-Glutamyl Transferase-to-Lymphocyte Ratio (GLR) among healthy controls (*n* = 70), patients with cirrhosis (*n* = 71), and patients with hepatocellular carcinoma (HCC) (*n* = 70). **A** SIRI was significantly elevated in both patient groups compared to controls, but did not differ between cirrhosis and HCC (median: 0.60 in controls, 1.20 in cirrhosis, 1.25 in HCC). **B** GLR demonstrated a marked stepwise increase from controls to cirrhosis to HCC, with a highly significant difference between all groups (median: 4.8 in controls, 15.0 in cirrhosis, 30.0 in HCC). Boxes represent the interquartile range (IQR), the line inside the box is the median, and whiskers extend to 1.5 × IQR

Both novel inflammatory indices demonstrated significant dysregulation across the study groups, with distinct patterns for SIRI and GLR (Table 4; Fig. 2).

The Systemic Inflammation Response Index (SIRI) was significantly elevated in both patient groups compared to healthy controls (median: 1.25 in HCC and 1.20 in cirrhosis vs. 0.60 in controls; *p* < 0.001). However, there was no significant difference in SIRI between the HCC and cirrhosis groups (*p* = 0.42). In contrast, the Gamma-Glutamyl Transferase-to-Lymphocyte Ratio (GLR) exhibited a marked stepwise increase, with median values of 30.0 in the HCC group, 15.0 in the cirrhosis group, and 4.8 in controls (*p* < 0.001). Critically, GLR was significantly higher in HCC patients compared to those with cirrhosis alone (*p* < 0.001).

These findings underscore a progressive systemic inflammatory and immunometabolic imbalance accompanying hepatocarcinogenesis, with GLR demonstrating a more pronounced discriminatory pattern between cirrhosis and HCC than SIRI.

### Diagnostic performance of inflammatory indices

**Fig. 3:**
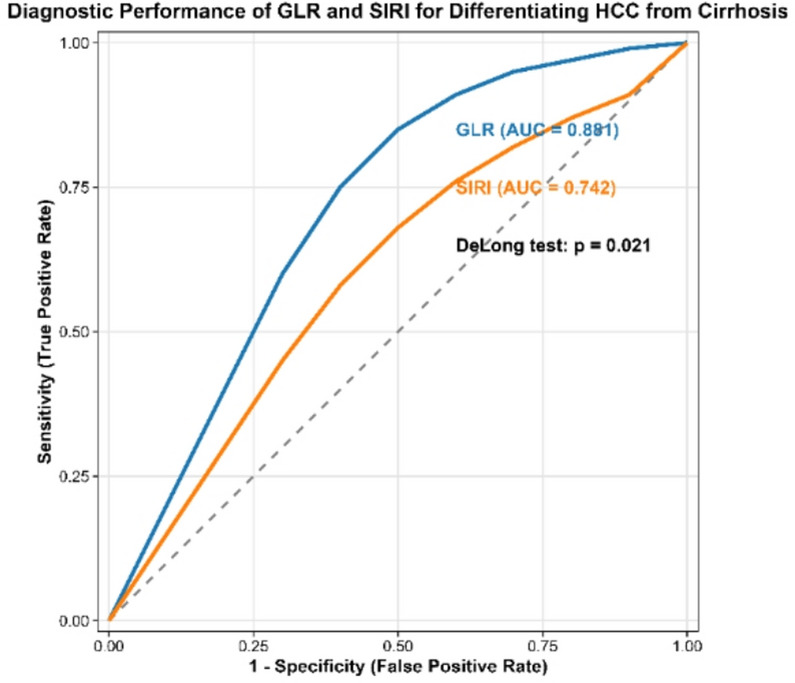
Diagnostic performance of GLR and SIRI for Differentiating HCC from Cirrhosis. Receiver operating characteristic (ROC) curves comparing the performance of GLR and SIRI in discriminating hepatocellular carcinoma (HCC) (*n* = 70) from liver cirrhosis (*n* = 71). The Gamma-Glutamyl Transferase-to-Lymphocyte Ratio (GLR, blue line) achieved a significantly higher area under the curve (AUC = 0.881) than the Systemic Inflammation Response Index (SIRI, red line; AUC = 0.742) (DeLong test, *p* = 0.021). The dashed grey line represents the line of no discrimination (AUC = 0.5)

Receiver-operating-characteristic (ROC) analysis was employed to evaluate the diagnostic utility of SIRI and GLR (Table 5; Fig. 3). For the critical discrimination between HCC and cirrhosis, GLR demonstrated strong discriminatory performance, with an area under the curve (AUC) of 0.881 (95% CI 0.842–0.921). At an optimal cut-off value of > 22.5, GLR achieved a sensitivity of 85.7% and a specificity of 81.6%. In contrast, SIRI showed moderate discriminative ability for this comparison (AUC = 0.742). A formal comparison confirmed that GLR showed significantly higher diagnostic accuracy compared to SIRI for distinguishing HCC from cirrhosis (DeLong test, *p* = 0.021). It is important to note that while a statistically optimal SIRI cut-off of > 1.18 was identified, its clinical applicability for this specific discrimination is constrained by the substantial overlap in SIRI values between the HCC and cirrhosis groups, as reflected in their nearly identical medians (1.25 vs. 1.20, *p* = 0.42).

**Table 5:** ROC analysis of SIRI and GLR for discrimination between study groups

Comparison	Index	AUC (95% CI)	Cut-off	Sensitivity (%)	Specificity (%)	*p*-value
HCC vs. Cirrhosis	**SIRI**	0.742 (0.671–0.813)	> 1.18	77.1	69.0	< 0.001
	**GLR**	0.881 (0.842–0.921)	> 22.5	85.7	81.6	< 0.001
Patients vs. Controls	**SIRI**	0.864 (0.825–0.903)	> 0.85	82.3	84.4	< 0.001
	**GLR**	0.935 (0.906–0.964)	> 14.2	89.1	90.0	< 0.001

Optimal cut-off values were determined using the Youden index and are exploratory, derived from this specific cohort. The difference between the AUC of GLR and SIRI for distinguishing HCC from cirrhosis was statistically significant (*p* = 0.021, DeLong test). Note that the SIRI cut-off (> 1.18) lies between the nearly identical medians of the HCC and cirrhosis groups (1.25 vs. 1.20), limiting its clinical utility for this discrimination

*Abbreviations*: *AUC* area under the receiver operating characteristic curve, *CI* confidence interval, *SIRI* Systemic Inflammation Response Index, *GLR* Gamma-Glutamyl Transferase-to-Lymphocyte Ratio

Both indices were highly effective in separating all patients (HCC and cirrhosis combined) from healthy controls, with GLR again achieving the highest accuracy (AUC = 0.935).

### Correlations between inflammatory and biochemical parameters

**Fig. 4:**
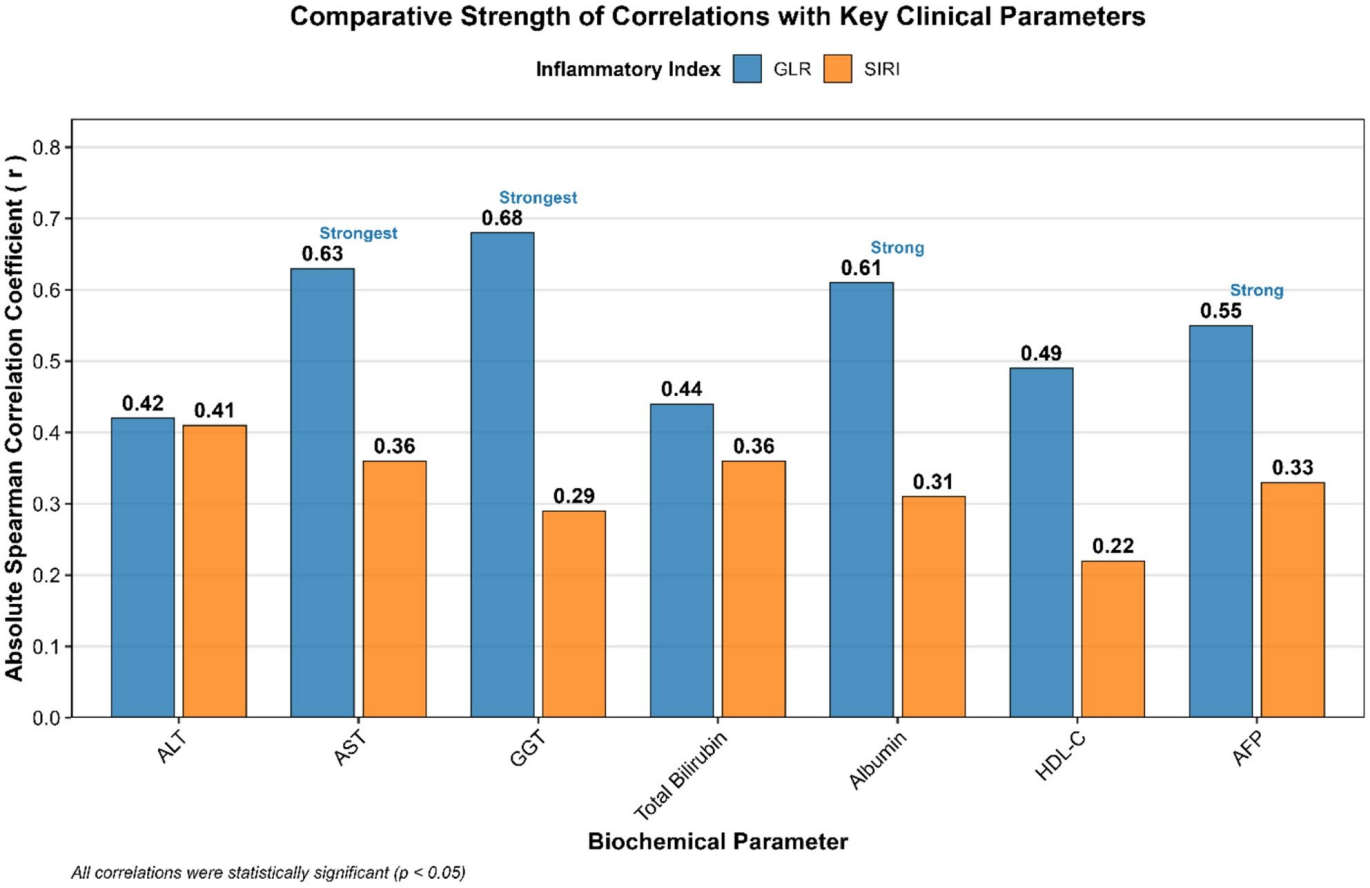
Comparative strength of correlations for GLR and SIRI with key clinical parameters. Bar chart comparing the magnitude of Spearman’s correlation coefficients (absolute r-value) for GLR (blue) and SIRI (red) with selected biochemical parameters. Analyses included all participants (*n* = 211). GLR demonstrates significantly stronger correlations across all parameters, particularly with AST, GGT, Albumin, and AFP, underscoring its closer association with markers of hepatic injury, synthetic function, and tumor burden. Error bars are not applicable as the graph displays fixed correlation coefficients. All correlations shown were statistically significant (*p* < 0.05)

Spearman correlation analysis revealed robust associations between the inflammatory indices and key biochemical parameters, with GLR demonstrating consistently stronger correlations than SIRI (Table 6; Fig. 4).

**Table 6:** Spearman correlation coefficients between inflammatory indices and selected biochemical parameters

Parameter	Correlation with GLR (*r*, *p*)	Correlation with SIRI (*r*, *p*)
ALT	0.42, < 0.001	0.41, < 0.001
AST	0.63, < 0.001	0.36, 0.002
GGT	0.68, < 0.001	0.29, 0.011
Total bilirubin	0.44, < 0.001	0.36, 0.002
Albumin	−0.61, < 0.001	−0.31, 0.008
HDL-C	−0.49, < 0.001	−0.22, 0.047
AFP	0.55, < 0.001	0.33, 0.004

Correlation strength was interpreted according to common guidelines: |r| < 0.3, weak; 0.3 ≤ |r| < 0.7, moderate; |r| ≥ 0.7, strong

*Abbreviations*: *ALT* alanine aminotransferase, *AST* aspartate aminotransferase, *GGT* gamma-glutamyl transferase, *HDL-C* high-density lipoprotein cholesterol, *AFP* alpha-fetoprotein

GLR showed strong positive correlations with markers of hepatocellular injury (AST, *r* = 0.63), cholestasis (GGT, *r* = 0.68), and tumor burden (AFP, *r* = 0.55) (all *p* < 0.001). It was also strongly inversely correlated with markers of synthetic liver function (albumin, *r* = -0.61) and metabolic health (HDL-C, *r* = -0.49). In contrast, SIRI demonstrated only low-to-moderate correlations with the same parameters, with its strongest associations being with markers of hepatocellular injury (ALT, *r* = 0.41) and bilirubin (*r* = 0.36).

This distinct correlation profile underscores that GLR, by integrating a hepatic enzyme (GGT) with an immune cell count (lymphocytes), more comprehensively reflects the intertwined pathways of hepatic dysfunction, metabolic dysregulation, and oncogenesis than SIRI, which is derived solely from myeloid and lymphoid cell counts.

### Univariate predictors of HCC

To inform the selection of variables for the multivariable diagnostic model, univariate logistic regression was performed comparing HCC and cirrhosis patients. Variables with *p* < 0.10 are presented in Table 7. Based on clinical relevance and the aim to capture distinct pathophysiological domains, GLR, AFP, albumin, and HDL-C were selected for the final multivariable model.

**Table 7:** Univariate logistic regression analysis of candidate predictors for hepatocellular carcinoma (HCC) versus cirrhosis.

Variable	OR (95% CI)	*p*-value	Included in Final Model?
GLR (per 5-unit increase)	1.32 (1.18–1.47)	< 0.001	Yes
AFP (log-transformed)	2.45 (1.88–3.20)	< 0.001	Yes
Albumin (g/dL)	0.42 (0.28–0.63)	< 0.001	Yes
HDL-C (mg/dL)	0.85 (0.80–0.91)	< 0.001	Yes
Platelets (×10⁹/L)	0.99 (0.98–1.00)	0.120	No
ALT (U/L)	1.01 (1.00–1.02)	0.085	No
AST (U/L)	1.02 (1.01–1.03)	0.002	No
Total Bilirubin (mg/dL)	1.25 (1.05–1.49)	0.012	No
Child–Pugh Class (B/C vs. A)	1.85 (1.20–2.85)	0.006	No
Ascites (Yes vs. No)	1.30 (0.85–2.00)	0.230	No

*Abbreviations*: *OR* Odds Ratio, *CI* Confidence Interval

Only variables with *p* < 0.10 are shown

### Composite diagnostic modeling and predictive value of integrated parameters

**Fig. 5:**
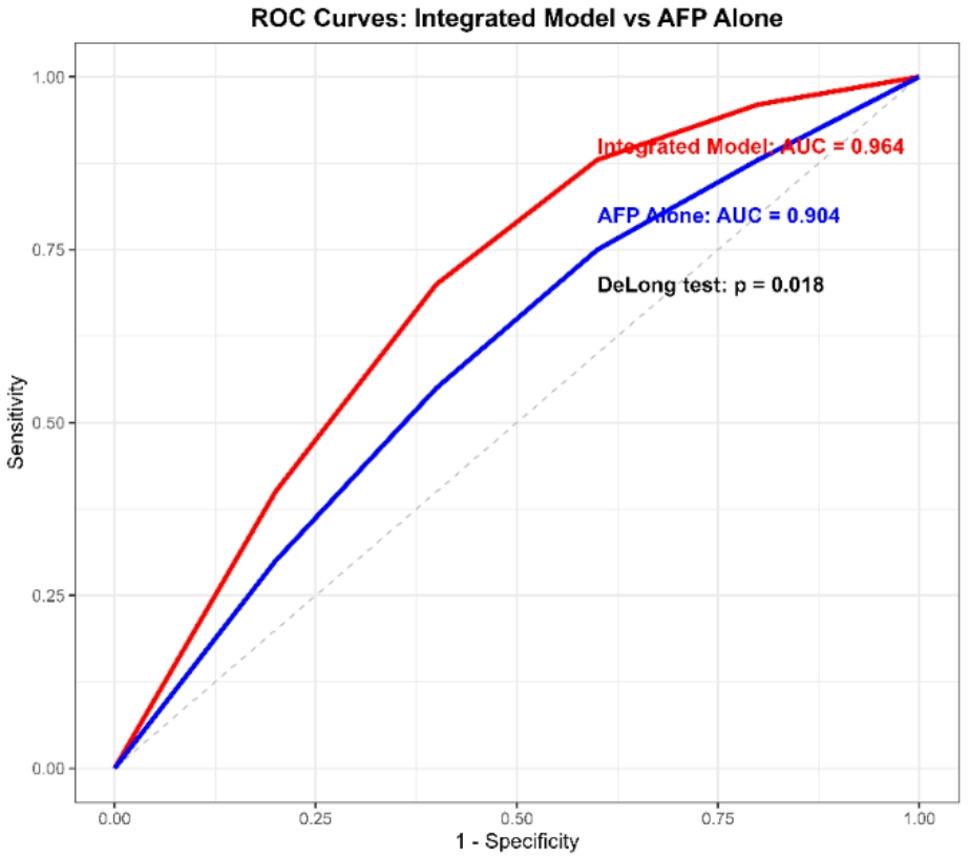
ROC curves for the integrated diagnostic model versus AFP alone. Comparison of the diagnostic performance for detecting HCC versus cirrhosis (HCC *n*=70, Cirrhosis *n*=71). The integrated multivariable model (red line), which includes GLR, AFP, and albumin, demonstrates strong discriminatory performance with an area under the curve (AUC) of 0.964. This performance is significantly superior to that of alpha-fetoprotein (AFP) alone (blue line, AUC = 0.904; DeLong test, *p* = 0.018)

To develop a robust diagnostic tool, a multivariable logistic regression model was constructed to predict HCC presence. The model incorporated the most promising novel index, GLR, alongside established biomarkers of liver function and tumor burden (albumin, AFP) and metabolic health (HDL-C) (Table 8, Fig. 5).

**Table 8:** Multivariate logistic regression and ROC performance of the integrated diagnostic model

Variable	β Coefficient	SE	Wald	*p*-value	OR (95% CI)
GLR	0.84	0.19	19.6	< 0.001	2.31 (1.59–3.34)
AFP	0.71	0.17	17.3	< 0.001	2.03 (1.46–2.91)
Albumin	−0.62	0.21	8.9	0.003	0.54 (0.34–0.83)
HDL-C	−0.39	0.22	3.1	0.078	0.68 (0.44–1.04)

The model was developed to predict HCC (*n* = 70) versus cirrhosis (*n* = 71). The events per variable (EPV) ratio was 17.5. Model performance: Apparent AUC = 0.964 (95% CI 0.942–0.987); optimism-corrected AUC via 500 bootstrap iterations = 0.942 (95% CI 0.915–0.968). Model calibration: Hosmer-Lemeshow goodness-of-fit test, *p* = 0.61

*Abbreviations*: *SE* standard error, *OR* odds ratio, *CI* confidence interval, *GLR* Gamma-Glutamyl Transferase-to-Lymphocyte Ratio, *AFP* alpha-fetoprotein, *HDL-C* high-density lipoprotein cholesterol

In the final model, elevated GLR (OR = 2.31, 95% CI 1.59–3.34) and elevated AFP (OR = 2.03, 95% CI 1.46–2.91) were significant independent predictors of HCC, while low serum albumin (OR = 0.54, 95% CI 0.34–0.83) was also independently associated with the outcome (Table 8). HDL-C did not retain independent significance (*p* = 0.078).

The integrated model demonstrated high apparent discriminative ability, achieving an area under the curve (AUC) of 0.964 (95% CI 0.942–0.987). After internal bootstrap validation, the optimism-corrected AUC was 0.942 (95% CI 0.915–0.968). This was significantly superior to the performance of AFP alone (AUC = 0.904; DeLong test, p = 0.018). The number of HCC events per predictor variable (EPV) was 17.5 (70 events / 4 predictors), supporting model stability. The model was well-calibrated (Hosmer–Lemeshow test, *p* = 0.61).

### Subgroup analysis of GLR performance

**Fig. 6:**
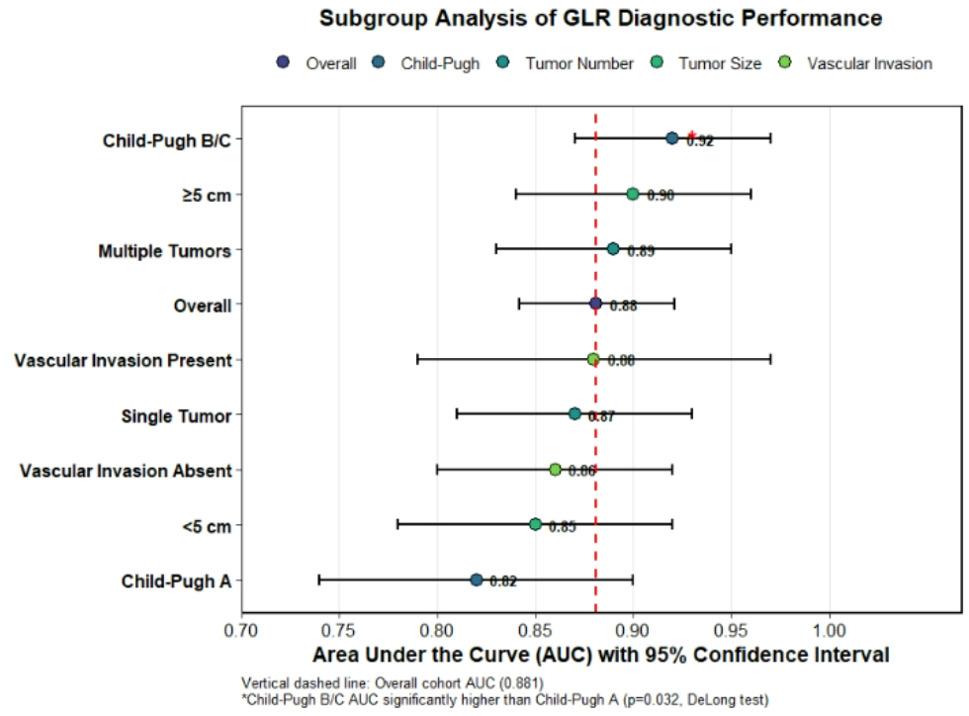
Subgroup analysis of GLR diagnostic performance. Forest plot illustrating the area under the curve (AUC) and 95% confidence intervals for the Gamma-Glutamyl Transferase-to-Lymphocyte Ratio (GLR) in discriminating hepatocellular carcinoma (HCC) from cirrhosis across various patient subgroups (overall HCC *n*=70, Cirrhosis *n*=71). The vertical dashed line represents the overall cohort AUC (0.881). The performance of GLR was significantly enhanced in patients with more advanced liver dysfunction (Child-Pugh Class B/C). Differences between subgroup AUCs were compared using the DeLong test

To assess the robustness and clinical applicability of the Gamma-Glutamyl Transferase-to-Lymphocyte Ratio (GLR), we conducted a subgroup analysis evaluating its diagnostic performance for distinguishing HCC from cirrhosis across key patient strata defined by liver disease severity and tumor characteristics (Table 9, Fig. 6).

**Table 9:** Diagnostic performance of GLR (> 22.5) for discriminating HCC from cirrhosis across patient subgroups

Subgroup	*N* (HCC/Cirrhosis)	AUC (95% CI)	Sensitivity (%)	Specificity (%)
Overall	70 / 71	0.881 (0.842–0.921)	85.7	81.6
By Child–Pugh Class				
Class A	47 / 39	0.82 (0.74–0.90)	80.9	79.5
Class B/C	23 / 32	0.92 (0.87–0.97)	91.3	84.4
By Tumor Number				
Single	45 / 71	0.87 (0.81–0.93)	84.4	81.6
Multiple	25 / 71	0.89 (0.83–0.95)	88.0	81.6
By Max Tumor Diameter				
<5 cm	38 / 71	0.85 (0.78–0.92)	81.6	81.6
≥5 cm	32 / 71	0.90 (0.84–0.96)	90.6	81.6
By Vascular Invasion				
Absent	58 / 71	0.86 (0.80–0.92)	84.5	81.6
Present	12 / 71	0.88 (0.79–0.97)	91.7	81.6

AUC for Child–Pugh B/C was significantly higher than for Child–Pugh A (*p* = 0.032, DeLong test). This comparison was pre-specified; other subgroup differences are presented descriptively

Table 9 presents the AUC, sensitivity, and specificity of GLR (>22.5) within these subgroups. GLR maintained strong discriminatory ability across all subgroups (AUCs 0.82–0.92). Notably, its performance was most robust in HCC patients with more advanced disease. The highest AUC was observed in the subgroup of patients with Child–Pugh class B/C cirrhosis (AUC = 0.92, 95% CI 0.87–0.97), significantly outperforming its performance in the Child–Pugh A subgroup (AUC = 0.82, DeLong test, *p* = 0.032). Furthermore, GLR demonstrated excellent diagnostic accuracy in patients with larger tumor burdens, such as those with a single tumor ≥5 cm or the presence of multiple tumors (AUC = 0.90 and 0.89, respectively).

### Survival analysis based on GLR stratification

Given the established link between systemic inflammation and cancer prognosis, we investigated the association between GLR and overall survival (OS) in the HCC cohort. Based on the optimal diagnostic cut-off value of 22.5, HCC patients were stratified into two groups: Low-GLR (≤22.5, n=28) and High-GLR (>22.5, *n*=42). Follow-up time was calculated from diagnosis to death or last clinical contact, with surviving patients censored at last follow-up. The median follow-up time for the entire HCC cohort was 18 months (interquartile range [IQR]: 12–24 months), estimated using the reverse Kaplan–Meier method. Over this period, there were 31 recorded deaths (44.3%) (Table 10).

**Table 10:** Univariable and multivariable Cox regression analysis for overall survival in the HCC cohort (*n* = 70)

Variable	Category	Unadjusted HR (95% CI)	*p*-value	Adjusted HR (95% CI)	*p*-value
GLR	Per 1-unit increase	1.06 (1.03–1.09)	< 0.001	1.05 (1.02–1.08)	0.001
GLR	> 22.5 vs. ≤22.5	3.45 (1.75–6.80)	< 0.001	2.95 (1.42–6.13)	0.004
Age	Per 1-year increase	1.02 (0.98–1.06)	0.301	1.01 (0.97–1.05)	0.551
Child-Pugh Class	B/C vs. A	2.75 (1.41–5.36)	0.003	2.48 (1.21–5.09)	0.013
AFP (ng/mL)	> 400 vs. ≤400	2.55 (1.35–4.82)	0.004	2.10 (1.05–4.18)	0.035
Tumor Burden*	High vs. Low	2.30 (1.18–4.49)	0.014	1.82 (0.89–3.71)	0.099

*High Tumor Burden was defined as the presence of any one of the following established adverse prognostic features: multiple tumors (regardless of individual size), macrovascular invasion, or a maximum tumor diameter ≥ 5 cm. This composite definition is consistent with major HCC staging systems (e.g., BCLC) and reflects aggressive tumor biology. Abbreviations: HR, Hazard Ratio; CI, Confidence Interval. Continuous GLR was modeled per a 1-unit increase to reflect the hazard associated with a clinically relevant incremental change

**Fig. 7:**
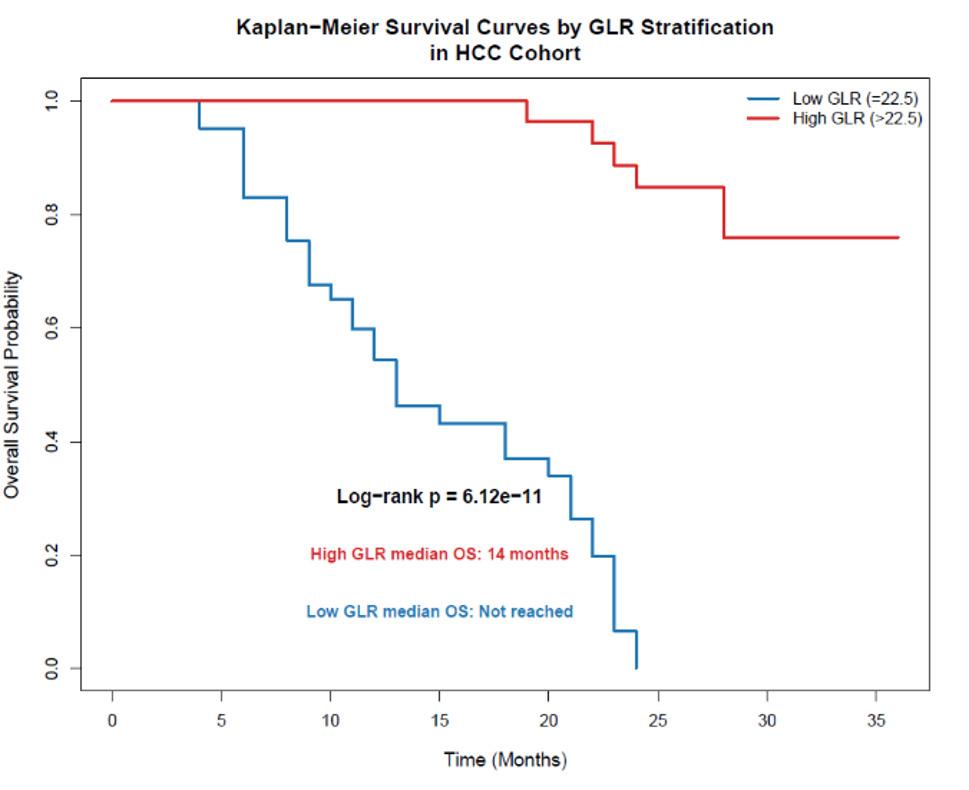
Kaplan-meier survival curves by GLR stratification in the HCC Cohort. Overall survival of patients with hepatocellular carcinoma (HCC) (*n*=70) stratified by the Gamma-Glutamyl Transferase-to-Lymphocyte Ratio (GLR) using the cut-off value of 22.5 (High-GLR, *n*=42; Low-GLR, *n*=28). Patients with a high GLR (>22.5) had a significantly shorter median overall survival (14 months) compared to those with a low GLR (≤22.5), for whom the median survival was not reached (Log-rank test, *p* < 0.001)

Kaplan-Meier survival analysis revealed a significant disparity in outcomes between the two groups (Fig. 7). Numbers at risk over time are provided in Table 11. Patients in the High-GLR group had a markedly poorer median OS of 14 months, compared to a median OS that was not reached in the Low-GLR group (Log-rank test, *p* < 0.001).

**Table 11:** Number of patients at risk over time by GLR group

Time (months)	Low-GLR (≤ 22.5)	High-GLR (> 22.5)
0	28	42
6	25	30
12	20	18
18	15	10
24	10	4

To determine the independent prognostic value of GLR, a Cox proportional hazards model was constructed, adjusting for age, Child-Pugh class, AFP (>400 ng/mL), and tumor burden (categorized as high if multiple tumors, vascular invasion, or maximum tumor diameter ≥5 cm were present). In this multivariable analysis (Table 10), a high GLR remained a significant independent predictor of mortality (Adjusted Hazard Ratio [HR] = 2.95, 95% CI 1.42–6.13; p = 0.004), alongside AFP >400 ng/mL (HR = 2.10, 95% CI 1.05–4.18; p = 0.035) and Child-Pugh class B/C (HR = 2.48, 95% CI 1.21–5.09; p = 0.013). When modeled as a continuous variable (per 1-unit increase), GLR remained significantly associated with mortality (adjusted HR = 1.05, 95% CI 1.02–1.08, p = 0.001), confirming a dose–response relationship independent of the dichotomized cut-off.

## Discussion

This prospective, case-control study was conceived to dissect the complex interplay of hematologic, metabolic, and inflammatory dysregulation that characterizes the transition from health to HCV-related cirrhosis and ultimately to hepatocellular carcinoma (HCC) in a dedicated Egyptian cohort. Against the backdrop of Egypt’s high historical burden of HCV, once estimated to affect over 6% of the adult population [3, 4], and the evolving landscape with rising non-HCV etiologies [6, 7], we aimed to evaluate novel inflammatory indices. Our findings reveal a landscape of progressive systemic compromise, with the Gamma-Glutamyl Transferase-to-Lymphocyte Ratio (GLR) emerging as a promising biomarker. It demonstrated a marked stepwise elevation across the disease continuum, offered superior discrimination between cirrhosis and HCC compared to the Systemic Inflammation Response Index (SIRI), enhanced multivariable diagnostic models, and served as an independent predictor of overall survival. This work provides a cohesive, multi-system pathophysiological map within a high-risk population. While individual parameter changes confirm established pathophysiology, the novel insight lies in their integrated progression and, critically, in the performance of the composite index GLR. We posit GLR as a pragmatic, low-cost tool derived from this integrative analysis, with significant potential to refine diagnostic and prognostic stratification in clinical practice [13, 22].

Our study population was well-characterized and matched for age and sex (Table 1), ensuring that the observed differences are likely attributable to disease state rather than demographic confounders. The clinical profiles of the cirrhosis and HCC groups revealed distinct patterns of hepatic decompensation (Table 2). The greater proportion of patients with severe ascites in the HCC group (9/70, 12.9%) compared to the cirrhosis group (2/71, 2.8%) may reflect the additional burden of portal hypertension from tumor invasion or portal vein thrombosis. Conversely, the cirrhotic group exhibited a higher proportion of Child-Pugh class C patients, which was primarily driven by worse coagulopathy (higher INR) and a higher prevalence of hepatic encephalopathy rather than by ascites severity (Table 2). This finding underscores that HCC can be diagnosed even in patients with moderately preserved synthetic function, highlighting the need for biomarkers independent of conventional severity scores, a niche where GLR excelled.

The comprehensive laboratory profiles (Table 3, Fig. 1) paint a clear picture of escalating systemic injury. The profound thrombocytopenia and anemia observed in both patient groups are classic sequelae of portal hypertension and chronic disease-related marrow suppression [23]. The prognostic implications of thrombocytopenia in HCC extend beyond bleeding risk; it is increasingly viewed as a marker of a compromised tumor microenvironment and is associated with poorer survival [24, 25].

The graded decline in serum albumin and rise in INR are direct reflections of failing hepatic synthetic capacity. More notably, we documented significant metabolic disturbances, including a progressively atherogenic lipid profile and marked hyperinsulinemia. This pattern is consistent with the concept of “liver-induced metabolic syndrome,” where chronic inflammation and hepatocellular damage disrupt lipid homeostasis and promote insulin resistance [6, 7]. This metabolic milieu is actively pro-oncogenic and is consistent with fostering an environment rich in oxidative stress that could promote carcinogenesis.

A cornerstone finding of our study is the strong performance of GLR. While both SIRI and GLR were elevated in patient groups versus controls, only GLR demonstrated a significant stepwise increase from cirrhosis to HCC (Table 4, Fig. 2). This superior discriminatory power was confirmed by ROC analysis, where GLR achieved an AUC of 0.881 for distinguishing HCC from cirrhosis, significantly outperforming SIRI (AUC = 0.742) (Table 5, Fig. 3). This suggests a simple panel of routine tests could dramatically improve the pre-test probability for HCC, especially given the limited discriminatory power of AFP alone in our cohort, which aligns with its well-known reduced sensitivity for early-stage or small tumors [26].

Notably, the median GGT level was higher in the HCC group than in the cirrhosis group (84 U/L vs. 55 U/L, Table 3). This elevation, combined with a profound and significant lymphopenia in the HCC cohort (median absolute lymphocyte count 1.2 vs. 1.5 × 10⁹/L), synergistically drove the GLR higher. This marked lymphopenia in HCC decreases the GLR denominator, thereby amplifying the ratio’s value. This underscores that the discriminatory power of GLR lies not in any single component but in the synergistic integration of hepatic stress and immune dysfunction. The elevated GGT in HCC reflects underlying hepatic oxidative stress and cholestasis, processes known to be involved in hepatocarcinogenesis [26, 27]. Its combination with lymphopenia in the GLR formula thus captures a distinct immunometabolic shift characteristic of tumor progression. Indeed, the prognostic and diagnostic superiority of GLR over isolated GGT or lymphocyte counts has been reported in other cohorts [13, 14], supporting its role as a composite biomarker reflective of both hepatic stress and immune exhaustion.

The biological rationale for the superiority of GLR is profound and lies in its synthesis of two cardinal pathophysiological pathways. The numerator, gamma-glutamyl transferase (GGT), is more than a cholestatic marker; it is integral to extracellular glutathione metabolism, and its elevation signifies a state of heightened hepatic oxidative stress that can foster genomic instability and a pro-tumorigenic microenvironment [26, 27]. The denominator, the absolute lymphocyte count, serves as a crude but accessible proxy for systemic immune competence. Progressive lymphopenia in chronic liver disease and cancer reflects T-cell exhaustion and impaired immune surveillance, a critical permissive factor for hepatocarcinogenesis [8]. Thus, the GLR elegantly quantifies the convergence of a pro-oncogenic ‘seed and soil’ dynamic: a liver under oxidative stress (high GGT) paired with a weakened host defense (low lymphocytes). This integrated measure of the hepatic-immune axis explains its stronger diagnostic correlation and discriminatory power compared to indices derived solely from peripheral blood cell counts.

Thus, GLR elegantly quantifies this convergence: a numerator (GGT) representing the ‘seed’ of genomic instability and oncogenic pressure [27], and a denominator (lymphocyte count) representing the failure of the ‘soil’s immune surveillance [8]. This suggests that indices embedding liver-derived information (GLR) may better reflect processes unique to hepatic oncogenesis than blood-count-only indices [13]. Furthermore, the poor clinical utility of SIRI for discriminating HCC from cirrhosis is underscored by the near-identical median values and significant overlap in its distribution between groups (Table 4). Although an ROC-derived cut-off (> 1.18) was calculated, it sits between two highly similar medians, resulting in poor specificity and limiting its practical application for this purpose.

By combining these elements, GLR captures a dynamic reminiscent of the “soil and seed” model of hepatocarcinogenesis. We acknowledge that elevated GGT may reflect both tumor-related cholestasis and chronic hepatic oxidative stress, while lymphopenia can indicate immune suppression associated with malignancy as well as advanced liver disease itself. Our cross-sectional design cannot untangle the precise temporal sequence of these changes; longitudinal studies are needed to clarify whether GLR elevation precedes or follows tumor development. Furthermore, we infer that lymphopenia may reflect T‑cell exhaustion, but we lacked immunophenotyping data; total lymphocyte counts are a crude proxy, and future studies should evaluate specific lymphocyte subsets to define the underlying immune dysfunction more precisely. Despite these limitations, GLR’s value appears to extend beyond being a simple proxy for tumor size, a notion supported by our finding that GLR remained an independent predictor of survival even after adjusting for a composite measure of high tumor burden (adjusted HR = 2.95, p = 0.004, Table 10). This makes GLR a more nuanced indicator than SIRI, which reflects systemic inflammation but lacks organ‑specific context [11, 28]. Our findings align with a growing body of international literature [13, 29, 30] and extend this evidence by establishing its diagnostic supremacy in a direct comparison within a high‑risk Egyptian population, a cohort historically under‑represented in such biomarker studies [31]. The correlation analysis further cements GLR’s biological relevance (Table 6, Fig. 4). Its strong positive correlations with markers of hepatocellular injury (AST), cholestasis (GGT itself), and tumor burden (AFP), coupled with its strong inverse correlation with synthetic function (albumin), demonstrate that GLR is a sensitive integrator of multiple disease pathways. The moderate correlation between GLR and AFP indicates partial biological overlap while supporting non-redundancy; GLR likely reflects inflammatory–immune dysregulation, whereas AFP is more closely linked to tumor burden and cellular differentiation. This multifaceted nature is precisely why it contributed independently to a powerful diagnostic model. Univariate analysis (Table 7) confirmed that GLR, along with AFP, albumin, and HDL-C, were among the strongest predictors of HCC at the p < 0.10 threshold. When combined with AFP and albumin in a multivariable logistic regression, the integrated model achieved a high apparent discriminative ability (AUC = 0.964), which remained strong after optimism correction (bootstrap-corrected AUC = 0.942) and was significantly superior to AFP alone (Table 8, Fig. 5). While these results are promising, external validation is required before clinical implementation. Nevertheless, this suggests that a simple panel of routine tests could potentially improve the pre-test probability for HCC in high-risk surveillance.

The robustness of GLR was confirmed through subgroup analysis (Table 9, Fig. 6). Its diagnostic performance remained strong across all strata but was particularly potent in patients with more advanced liver dysfunction (Child-Pugh B/C, AUC = 0.92). This indicates that GLR retains its utility in the very population where clinical assessment is most challenging. Furthermore, its prognostic value was unequivocal (Table 10, Fig. 7). Follow-up was rigorously defined from diagnosis to death or last clinical contact, with median follow-up estimated via reverse Kaplan–Meier. Censoring patterns were clear, with decreasing numbers at risk over time as expected (Table 11). In our HCC cohort, both dichotomized (> 22.5) and continuous (per 1-unit increase) GLR were significant independent predictors of reduced overall survival (adjusted HR = 2.95 and 1.05, respectively), even after controlling for Child-Pugh class and AFP. This dose–response relationship underscores the robustness of GLR as a prognostic marker beyond a single cut-off.

Collectively, our data construct a coherent pathophysiological narrative. The journey from HCV infection to HCC is marked by a progressive, systemic unraveling. However, the critical transition to malignancy is most accurately associated with a biomarker that encapsulates the escalating hepatic oxidative stress and the concomitant erosion of adaptive immune surveillance. GLR, by its very design, captures this convergence.

This study provides several novel and significant contributions. It is one of the first prospective studies from Egypt to perform a comprehensive, head-to-head comparison of clinical, metabolic, and inflammatory parameters, including SIRI and GLR, across the entire health-cirrhosis-HCC spectrum. We move beyond merely validating GLR’s prognostic value to firmly establishing its superior diagnostic accuracy for the critical differentiation between cirrhosis and HCC in a high-burden population. The demonstration that a simple, inexpensive index can be seamlessly integrated into existing laboratory panels to significantly refine both diagnosis and prognosis offers an immediately actionable strategy for improving patient management in Egypt and similar healthcare landscapes.

Importantly, GLR is not proposed as a standalone screening test for hepatocellular carcinoma, which remains based on ultrasound as per international guidelines [2, 17]. Rather, it represents a readily available, low-cost adjunctive biomarker that may enhance risk stratification within established surveillance frameworks for patients with cirrhosis [13]. GLR is not intended to replace imaging-based diagnosis, but rather to serve as a laboratory-based risk stratification tool that reflects systemic pathophysiology and may complement existing surveillance and diagnostic modalities.In high-burden settings such as Egypt, where large numbers of patients with HCV-related cirrhosis require long-term monitoring [4, 30], GLR may help identify individuals at particularly elevated risk who may benefit from intensified surveillance strategies.

From a clinical standpoint, our findings provide a validated rationale for evaluating GLR in surveillance protocols for the large, historically high-risk cohort of Egyptian patients with HCV-related cirrhosis. This study was deliberately designed to establish a robust benchmark within this defined etiologic context. As the etiologic landscape evolves, future studies must assess the performance of GLR across other major etiologies, including the rising burden of MASLD. Our work provides the necessary foundational evidence to inform and justify those critical next steps in biomarker translation. For the research community, our findings necessitate external validation in larger, multicenter cohorts. Future studies should also investigate the dynamic nature of GLR; serial measurements may prove valuable in monitoring treatment response. Indeed, as Metabolic Dysfunction-Associated Steatotic Liver Disease (MASLD) is projected to become a leading cause of HCC in Egypt over the next decade, validating GLR across metabolic liver disease phenotypes, potentially building on dietary intervention models for MASLD, will be essential to ensure its long-term applicability [32].

We acknowledge several limitations. First, and most critically, this study was conducted exclusively on patients with HCV-related cirrhosis and HCC. While this deliberate etiologic homogeneity strengthens internal validity by eliminating confounding from divergent disease mechanisms (e.g., HBV, alcohol, MASLD), it simultaneously represents a significant disadvantage regarding generalizability. The inflammatory, metabolic, and hematologic profiles observed here may differ substantially in HCC arising from other etiologies. For instance, MASLD-related HCC often occurs in the context of metabolic syndrome, obesity, and diabetes, conditions we explicitly excluded, and may exhibit distinct inflammatory signatures. Therefore, our findings cannot be directly extrapolated to non-HCV populations without validation. This etiologic restriction should be carefully considered when interpreting our results and is a clear limitation of the present study. Future studies must evaluate GLR’s performance across the full spectrum of liver disease etiologies, particularly given the rising global burden of MASLD. Second, while we applied strict exclusion criteria to ensure etiologic homogeneity, unmeasured confounders, such as detailed histories of antiviral therapy or subtle metabolic comorbidities, cannot be entirely ruled out. Third, the reliance on radiological criteria for HCC diagnosis in most cases, while conforming to international guidelines, precludes correlating GLR with histopathological features like microvascular invasion or tumor differentiation. Fourth, the GLR cut-off (> 22.5) was derived from this cohort using the Youden index and should be considered exploratory until externally validated. Fifth, we explicitly and deliberately excluded patients who had received direct-acting antiviral (DAA) therapy within the preceding 12 months, and we wish to highlight this prominently. This decision was made intentionally to avoid confounding from the rapid immunological and biochemical changes that accompany viral clearance following successful DAA treatment. DAA therapy is known to induce significant alterations in lymphocyte subsets, reduce systemic inflammation, and improve liver function tests, all of which would directly impact the calculation and interpretation of SIRI and GLR. While this exclusion was methodologically necessary to ensure internal validity, it simultaneously represents an important limitation. Our results are directly applicable only to DAA-naïve patients or those with a remote treatment history (> 12 months). They do not reflect the post-DAA setting, where inflammatory and metabolic parameters may be substantially modified, and this should be acknowledged as a constraint on the applicability of our findings. This distinction is increasingly critical given Egypt’s successful national HCV treatment campaign, which has treated millions of patients with DAA therapy [33]. The growing population of patients with cirrhosis who have achieved sustained virologic response (SVR) represents a new and evolving cohort requiring dedicated study. We explicitly acknowledge that our findings do not address this population, and future research should specifically evaluate the dynamics of GLR and other inflammatory indices during and after DAA therapy, and determine whether these biomarkers retain their prognostic utility in patients who have cleared the virus. Such studies are essential for comprehensive risk stratification in the modern era of HCV management. Furthermore, applying this same derived cut-off for survival analysis within the cohort may introduce optimism; external validation of the prognostic threshold is therefore required. Although dichotomization facilitates clinical interpretation, continuous modeling (Table 10) confirmed a dose–response relationship, supporting GLR’s role as a robust prognostic marker. Finally, the follow-up duration, though sufficient to establish a strong prognostic signal, should be extended in future studies to assess GLR’s value in predicting long-term outcomes and recurrence. We propose that biomarker development in a biologically heterogeneous disease such as HCC may optimally follow a ‘precision-pathway’ model, wherein initial validation in etiologically homogeneous cohorts provides the biological clarity and statistical confidence necessary to guide subsequent evaluation in more heterogeneous, real-world populations.

Generalizability and the Critical Limitation of Etiologic Restriction. We wish to emphasize explicitly that this study was conducted exclusively on patients with HCV-related cirrhosis and HCC. This deliberate design choice was made to minimize confounding and provide a clear pathophysiological benchmark within a single, well-defined disease pathway. Given Egypt’s historically high burden of HCV, this focus remains clinically relevant for the local population. However, this etiologic restriction is a major disadvantage and limitation that significantly constrains the generalizability of our findings. The etiological landscape of chronic liver disease is evolving globally and within Egypt, with a rising prevalence of metabolic dysfunction-associated steatotic liver disease (MASLD), hepatitis B virus (HBV), alcohol-related liver disease, and other etiologies [32]. Each of these etiologies carries distinct pathogenic mechanisms, inflammatory profiles, and metabolic contexts that may differentially influence both the progression to HCC and the performance of biomarkers such as GLR. For example, MASLD-related HCC often develops in patients with obesity, insulin resistance, and diabetes—conditions we explicitly excluded—and may exhibit fundamentally different inflammatory and metabolic signatures. Therefore, our findings should not be directly extrapolated to non-HCV populations without validation. This etiologic homogeneity, while methodologically necessary for internal validity, is unequivocally a limitation of the study. Future studies are urgently needed to evaluate GLR’s diagnostic and prognostic performance across diverse etiologies, including MASLD, HBV, and mixed-etiology cohorts. Until such validation studies are completed, the clinical applicability of GLR remains confined to the HCV context in which it was developed.

We also acknowledge and emphasize that the exclusion of patients with recent DAA therapy, while methodologically necessary to avoid confounding, means our findings are confined to the DAA-naïve or remotely treated population. As Egypt transitions from high HCV prevalence to a largely DAA-treated population, validating these biomarkers in patients who have achieved sustained virologic response becomes an urgent priority. This limitation should be clearly recognized when considering the applicability of our results in contemporary clinical practice.

## Data Availability

All relevant data are included in this published article.

## References

[1] Sung H, Ferlay J, Siegel RL, Laversanne M, Soerjomataram I, Jemal A, Bray F. Global cancer statistics 2020: GLOBOCAN estimates of incidence and mortality worldwide for 36 cancers in 185 countries. Cancer J Clin. 2021;71(3):209–49. 10.3322/caac.21660 33538338

[2] Forner A, Reig M. Carcinoma Bruix JHepatocellular. Lancet. 2018;391(10127):1301–4. 29307467 10.1016/S0140-6736(18)30010-2

[3] El-Zanaty F, Way A. Egypt Demographic and Health Survey 2014: Ministry of Health. El-Zanaty and Associates, Cairo, Egypt, and Macro International, and Rockville, MD. 2015.

[4] Kandeel A, Genedy M, El-Refai S, Funk AL, Fontanet A, Talaat M. The prevalence of hepatitis C virus infection in Egypt 2015: implications for future policy on prevention and treatment. Liver Int. 2017;37(1):45–53. 27275625 10.1111/liv.13186 PMC5145777

[5] Gao B, Tsukamoto H. Inflammation in alcoholic and nonalcoholic fatty liver disease: friend or foe? Gastroenterology. 2016;150(8):1704–9. 26826669 10.1053/j.gastro.2016.01.025 PMC4887345

[6] Liu C, Liu T, Zhang Q, Jia P, Song M, Zhang Q, Ruan G, Ge Y, Lin S, Wang Z, Xie H. New-onset age of nonalcoholic fatty liver disease and cancer risk. JAMA Netw Open. 2023;6(9):e2335511. 37747732 10.1001/jamanetworkopen.2023.35511 PMC10520743

[7] Nishida N. Metabolic disease as a risk of hepatocellular carcinoma. Clin Mol Hepatol. 2020;27(1):87. 33317238 10.3350/cmh.2020.0302 PMC7820215

[8] Ringelhan M, Pfister D, O’Connor T, Pikarsky E, Heikenwalder M. The immunology of hepatocellular carcinoma. Nat Immunol. 2018;19(3):222–32. 29379119 10.1038/s41590-018-0044-z

[9] Kim KM, Shim SG, Sinn DH, Song JE, Kim BS, Kim HG, Child-Pugh MELD. MELD-Na, and ALBI scores: which liver function models best predicts prognosis for HCC patient with ascites? Scand J Gastroenterol. 2020;55(8):951–7. 32698637 10.1080/00365521.2020.1788139

[10] Dolan RD, McSorley ST, Horgan PG, Laird B, McMillan DC. The role of the systemic inflammatory response in predicting outcomes in patients with advanced inoperable cancer: systematic review and meta-analysis. Crit Rev Oncol/Hematol. 2017;116:134–46. 28693795 10.1016/j.critrevonc.2017.06.002

[11] Qi Q, Zhuang L, Shen Y, Geng Y, Yu S, Chen H, Liu L, Meng Z, Wang P, Chen Z. A novel systemic inflammation response index (SIRI) for predicting the survival of patients with pancreatic cancer after chemotherapy. Cancer. 2016;122(14):2158–67. 27152949 10.1002/cncr.30057

[12] Wei L, Xie H, Yan P. Prognostic value of the systemic inflammation response index in human malignancy: a meta-analysis. Medicine. 2020;99(50):e23486. 33327280 10.1097/MD.0000000000023486 PMC7738007

[13] Peng X, Huang Y, Zhang M, Chen Y, Zhang L, He A, Luo R. Prognostic and Clinical Significance of Aspartate Aminotransferase-to‐Lymphocyte Ratio Index in Individuals with Liver Cancer: A Meta‐Analysis. Dis Markers. 2022;2022(1):3533714. 35186165 10.1155/2022/3533714 PMC8850034

[14] Zhou PC, Huang R, Wang HT, Yang J, Peng JD, Fu ZX, Liao WJ, Ma HQ, Wu LQ, Li EL. Gamma-glutamyl transferase-to-lymphocyte ratio as a prognostic marker in patients with hepatocellular carcinoma undergoing hepatectomy. World J Gastrointest Surg. 2025;17(2):98578. 40061977 10.4240/wjgs.v17.i2.98578 PMC11886001

[15] Ali AA, Gamal SE, Anwar R, Elzahaf E, Eskandere D. Assessment of clinico-epidemiological profile of Hepatocellular carcinoma in the last two decades. Egypt J Intern Med. 2023;35(1):18.

[16] Ranković B, Hauptman N. Circulating microRNA panels for detection of liver cancers and liver-metastasizing primary cancers. Int J Mol Sci. 2023;24(20):15451. 37895131 10.3390/ijms242015451 PMC10607808

[17] Marrero JA, Kulik LM, Sirlin CB, Zhu AX, Finn RS, Abecassis MM, Roberts LR, Heimbach JK. Diagnosis, Staging, and Management of Hepatocellular Carcinoma: 2018 P ractice G uidance by the A merican A ssociation for the Study of Liver Diseases. Hepatology. 2018;68(2):723–50. 29624699 10.1002/hep.29913

[18] Abdelfatah AA, Youssef MM, Abdel-Wahab M, Gouida MS, El-Emshaty HM. Predictive Significance of the Inflammatory Activities and GGT in Hepatocellular Carcinoma Development. Mansoura J Chem. 2025;68(5):1–9.

[19] Pugh RN, Murray-Lyon IM, Dawson JL, Pietroni MC, Williams R. Transection of the oesophagus for bleeding oesophageal varices. Br J Surg. 1973;60(8):646–9. 4541913 10.1002/bjs.1800600817

[20] Youden WJ. Index for rating diagnostic tests. Cancer. 1950;3(1):32–5. 15405679 10.1002/1097-0142(1950)3:1<32::aid-cncr2820030106>3.0.co;2-3

[21] DeLong ER, DeLong DM, Clarke-Pearson DL. Comparing the areas under two or more correlated receiver operating characteristic curves: a nonparametric approach. Biometrics. 1988;1:837–45. 3203132

[22] Xu L, Yu S, Zhuang L, Wang P, Shen Y, Lin J, Meng Z. Systemic inflammation response index (SIRI) predicts prognosis in hepatocellular carcinoma patients. Oncotarget. 2017;8(21):34954. 28430597 10.18632/oncotarget.16865 PMC5471025

[23] Afdhal N, McHutchison J, Brown R, Jacobson I, Manns M, Poordad F, Weksler B, Esteban R. Thrombocytopenia associated with chronic liver disease. J Hepatol. 2008;48(6):1000–7. 18433919 10.1016/j.jhep.2008.03.009

[24] Pang Q, Qu K, Zhang JY, Song SD, Liu SS, Tai MH, Liu HC, Liu C. The prognostic value of platelet count in patients with hepatocellular carcinoma: a systematic review and meta-analysis. Medicine. 2015;94(37):e1431. 26376382 10.1097/MD.0000000000001431 PMC4635796

[25] Kraj L, Chmiel P, Gryziak M, Grabowska-Derlatka L, Szymański Ł, Wysokińska E. Impact of thrombocytopenia on survival in patients with hepatocellular carcinoma: updated meta-analysis and systematic review. Cancers. 2024;16(7):1293. 38610973 10.3390/cancers16071293 PMC11011012

[26] Whitfield JB. Gamma-glutamyl transferase. Crit Rev Clin Lab Sci. 2001;38(4):263–355. 11563810 10.1080/20014091084227

[27] Stark AA. Oxidative metabolism of glutathione by γ-glutamyl transpeptidase and peroxisome proliferation: the relevance to hepatocarcinogenesis. A hypothesis. Mutagenesis. 1991;6(4):241–5. 1682786 10.1093/mutage/6.4.241

[28] Zhang S, Tang Z. Prognostic and clinicopathological significance of systemic inflammation response index in patients with hepatocellular carcinoma: a systematic review and meta-analysis. Front Immunol. 2024;15:1291840. 38469315 10.3389/fimmu.2024.1291840 PMC10925676

[29] Li Z, Luo J, Peng L, Wu B, Yu Z. Prognostic significance of systemic immune-inflammation index in hepatocellular carcinoma: a meta-analysis. Clin Translational Oncol. 2025;19:1–6. 10.1007/s12094-025-04028-3 40828354

[30] Omran D, Alboraie M, Zayed RA, Wifi MN, Naguib M, Eltabbakh M, Abdellah M, Sherief AF, Maklad S, Eldemellawy HH, Saad OK. Towards hepatitis C virus elimination: Egyptian experience, achievements and limitations. World J Gastroenterol. 2018;24(38):4330. 30344418 10.3748/wjg.v24.i38.4330 PMC6189850

[31] El-Serag HB, Kanwal F. Epidemiology of hepatocellular carcinoma in the United States: where are we? Where do we go? Hepatology. 2014;60(5):1767–75. 24839253 10.1002/hep.27222 PMC4211957

[32] Elhoseeny MM, Rageh F, Bakry N, Elgamal R, Ahmed SS, Rezk SM, Othman AA. Culturally adapted hypocaloric diet improves hepatic steatosis, inflammatory and oxidative biomarkers in Egyptian MASLD patients: a single-arm interventional study. Lipids Health Dis. 2025;24(1):286. 40988029 10.1186/s12944-025-02710-7 PMC12455765

[33] Hafez RS, Semeya AA, Elgamal R, Othman AA. Direct-acting antiviral therapy reduces variceal rebleeding and improves liver function in hepatitis C virus-related cirrhosis: A multicenter retrospective cohort study. World J Hepatol. 2025;17(11):110638. 41368107 10.4254/wjh.v17.i11.110638 PMC12683348

